# Sub genome anchored physical frameworks of the allotetraploid Upland cotton (*Gossypium hirsutum* L.) genome, and an approach toward reference-grade assemblies of polyploids

**DOI:** 10.1038/s41598-017-14885-w

**Published:** 2017-11-10

**Authors:** Christopher A. Saski, Brian E. Scheffler, Amanda M. Hulse-Kemp, Bo Liu, Qingxin Song, Atsumi Ando, David M. Stelly, Jodi A. Scheffler, Jane Grimwood, Don C. Jones, Daniel G. Peterson, Jeremy Schmutz, Z. Jeffery Chen

**Affiliations:** 1 Institute of Translational Genomics, Clemson University, Clemson, SC USA; 2 USDA-ARS, Genomics and Bioinformatics Research Unit, Stoneville, MS USA; 3 Department of Soil and Crop Sciences, Texas A&M University, College Station, TX USA; 4 Department of Molecular Biosciences, Center for Computational Biology and Bioinformatics, and Institute for Cellular and Molecular Biology, University of Texas, Austin, TX 78712 USA; 5 USDA-ARS, Crop Genetics Research Unit, Stoneville, MS USA; 6 Agriculture and Environmental Research, Cotton Incorporated, Cary, NC USA; 7 Institute for Genomics, Biocomputing & Biotechnology and Department of Plant & Soil Sciences, Mississippi State University, Mississippi State, MS USA; 8 HudsonAlpha Institute for Biotechnology, Huntsville, AL USA

## Abstract

Like those of many agricultural crops, the cultivated cotton is an allotetraploid and has a large genome (~2.5 gigabase pairs). The two sub genomes, A and D, are highly similar but unequally sized and repeat-rich, which pose significant challenges for accurate genome reconstruction using standard approaches. Here we report the development of BAC libraries, sub genome specific physical maps, and a new-generation sequencing approach that will lead to a reference-grade genome assembly for Upland cotton. Three BAC libraries were constructed, fingerprinted, and integrated with BAC-end sequences (BES) to produce a *de novo* whole-genome physical map. The BAC map was partitioned by sub genomes through alignment to the diploid progenitor D-genome reference sequence with densely spaced BES anchor points and computational filtering. The physical maps were validated with FISH and genetic mapping of SNP markers derived from BES. Two pairs of homeologous chromosomes, A11/D11 and A12/D12, were used to assess multiplex sequencing approaches for completeness and scalability. The results represent the first sub genome anchored physical maps of Upland cotton, and a new-generation approach to the whole-genome sequencing, which will lead to the reference-grade assembly of allopolyploid cotton and serve as a general strategy for sequencing other polyploid species.

## Introduction

Upland cotton (*Gossypium hirsutum* L.), the most extensively cultivated cotton species worldwide, is the most important renewable textile fiber and a significant oilseed crop^1^. The genus *Gossypium* includes ~45 diploid (2n = 2x = 26) and seven tetraploid (2n = 4x = 52) species. Allotetraploids arose in the New World from an interspecific hybridization event between an A-genome-like African species and a D-genome-like American species^2,3^, which occurred ~1-2 million years ago^4^. The closest relatives of these allopolyploid progenitors are the A-genome species *Gossypium herbaceum* L. (A_1_) and *Gossypium arboreum* L. (A_2_)^5^ and the D-genome species *Gossypium raimondii* (D_5_) Ulbrich^6^. The A-genome species produce spinnable fiber and are cultivated on a limited scale, whereas the D-genome fiber is rudimentary and not useful^7^. The fiber in allotetraploids is much longer and stronger, suggesting activation and/or silencing of homeologous fiber-related genes by genetic and epigenetic mechanisms^8–10^. Only two of the seven allotetraploid species, *Gossypium hirsutum* L. and *Gossypium barbadense* L. are domesticated and cultivated. Upland cotton (*G. hirsutum* L.) is indigenous to Central and South America, the Caribbean, and numerous islands in the Atlantic and Pacific oceans^4,11^. Most production (>95%) in the USA is Upland cotton, and the remainder is Pima cotton (*G. barbadense* L.) with extra-long fiber suitable for high quality textiles. Generating a quality reference assembly will be a critical foundation to advancing these species.

To date, approximately 77 plant genomes from 74 viridiplantae species have been sequenced (Phytozome release v12.1). These include reference grade assemblies in paleopolyploid species such as maize^12^, soybean^13^, and tomato^14^, but few allopolyploid crop genomes have been sequenced. Reference grade assemblies can be defined by long, contiguous contigs that have been accurately placed by a dense genetic/cytogenetic map into pseudomolecule representations of the chromosome. Draft quality assemblies are typically highly fragmented and contain an abundance of unplaced contigs and scaffolds. The currently available draft allopolyploid assemblies are highly fragmented consisting of short contigs and scaffolds with a significant portion of the genome missing or not anchored to chromosomes. For example, the scaffold N_50_ for *Brassica napus* ^15^ is 764 kilo-base pairs (Kb), 345–386 Kb for *Nicotiana tabacum* ^16^, and 515-4,297 base pairs (bp) (contigs) for wheat^17^.

Draft genome sequences have been reported recently for several *Gossypium* species, including *G. raimondii* (D_5_)^6,18^, *G. arboreum* (A_2_)^5^, *G. barbadense* (AD_2_)^19^, and *G. hirsutum* (AD_1_)^20,21^. The D_5_ diploid progenitor genome is smallest (800 megabase pairs, Mb) and has the highest quality genome assembly, with a scaffold N_50_ of 18.8 Mb^6^. The A_2_ genome is twice the size, and its assembled sequence has shorter scaffolds (N_50_ = 666 Kb) with an overabundance of unscaffolded contigs^5^. Assembly statistics for both of the available *G. hirsutum* draft assemblies suggests a high degree of incompleteness and lack of contiguity (scaffold N_50_ = 1,600 Kb)^21^, (N_50_ = 764 Kb)^20^, implying that the existing strategies to sequencing allopolyploid genomes cannot fully resolve heterozygous, paralogous and homeologous genes and repetitive DNA elements. Because of these challenges, generalized progressions in the development of trait genetics and tools for understanding polyploid species has relied on exploiting the extant progenitor or progenitor-related species as a precursor, and utilize that data to make inferences and insights toward the polyploid. This is obvious in cotton^6,7,22^ and other polyploid species, such as *Brassica napus* ^15,23^, and coffee^24^. However, the subsequent production of polyploid genome assemblies has typically not utilized the progenitor assemblies. The development of an interface, such as aligned BACs between the extant diploid sequence(s) and the polyploid assembly would be a novel tool in dissecting, tracking and understanding polyploid events.

BAC-based whole-genome physical mapping and hierarchal BAC-by-BAC sequencing techniques have served as the portal approach to reference grade genome assemblies for complex plant species, such as *Arabiodopsis* ^25^, rice^26^, maize^27^, and peach^28^. Modern assembly algorithms such as ABySS^29^, CABOG^30^, SoapDenovo^31^, etc., are typically confounded by repeat rich, complex plant genomes, especially when multiple sub genomes are present. Hierarchical BAC sequencing offers a format to readily partition the genome into manageable sized segments for local assemblies. This approach has served as the gold standard for genome sequencing, but is very time-consuming, expensive, and has yet to be applied in polyploid crop species in a whole-genome manner. The steady decline in per-base sequencing costs has promoted many shotgun-style genome sequencing efforts over the last several years, but the utility of these fragmented assemblies in both biology and breeding applications has been challenged in comparison to species endowed with a reference-grade quality sequence.

Here we developed a new-generation hierarchical BAC-by-BAC approach, integrated with emerging sequencing technologies to provide a reference-grade quality framework for the genome assembly of *Gossypium hirsutum* (var. TM-1). This approach takes advantage of the majority of the available cotton genomic resources produced to date, and the cost effectiveness and speed of second and third generation sequencing technologies; while maintaining the precision of traditional physical mapping and hierarchical sequencing approaches and the reference-grade quality of assembled products. The Upland cotton physical map was assembled *de novo* and subsequently partitioned into A and D sub genomes, which were comparatively analyzed using available D-genome and A- and D-sub genome sequences. Validation and utility of the physical map were tested using two homeologous chromosome pairs A11/D11 and A12/D12, which harbor important agronomic and morphological traits, including resistance to diseases and pathogens, male sterility, glanding, presence of nectaries and naked seed. Sequencing approaches using different-size pools of minimum tiling path (MTP) BACs have provided useful insights into future sequencing and assembling of the complete allotetraploid cotton genome, which will produce a reference-grade genome sequence for cotton and other polyploid species.

## Results

### TM-1 BAC library construction and characterization

We constructed three high-quality BAC libraries of Upland cotton (*G. hirsutum* L. acc. Texas-Marker 1, TM-1) with complementary genome fragmentation approaches. Two BAC libraries are composed of clones with inserts derived from partial restriction digestion (Gh_TBh and GH_TBb), and the third is derived of inserts resulting from mechanical genome fractionation (Gh_TBr) (Lucigen, Madison, Wisconsin). The Gh_TBh and Gh_TBb BAC libraries consist of 50,304 clones each; with an average insert size of 160 Kb and 150 Kb, respectively (Supplementary Table 1, Supplementary Figure 1 and 2A,B). The Gh_TBr random-sheared BAC library consists of ~160,000 clones with an average insert size of ~100 Kb (Supplementary Table 1, Supplementary Figure 2C). Based on an estimated genome size of ~2.5 gigabases (Gb)^32^, the combined BAC resources represent approximately 10-genome equivalents. These BAC resources are publically available at the Clemson University Genomics Institute (www.genome.clemson.edu).

### High Information Content Fingerprinting and BAC-end sequencing

High Information Content Fingerprinting (HICF)^33^ was used to assemble overlapping BAC clones into contigs. A total of 103,680 BACs (88% comprised of restriction-derived BACs) were subject to HICF. After applying stringent post-processing filters, the final validated BAC fingerprint dataset consisted of 90,083 BACs. The restriction-derived BAC set resulted in an average of 122 bands per clone; while the random sheared BACs resulted in an average of 75 bands per clone (Supplementary Table 2). A consensus band (CB) size for the BAC fingerprinting products was estimated to be 1,305 bp, and the collective validated dataset corresponds to ~6X genome coverage, with an estimated haploid *G. hirsutum* TM-1 genome size of 2.5 Gb. Each fingerprinted BAC was subject to insert end sequencing, which after quality filtering resulted in a total of 179,209 Sanger BAC-end sequence pairs with a mean high-quality read length of 576 bp^21^. These BAC-end sequences account for approximately 0.05X genomic coverage and by integration with the BAC fingerprints serve as sequence anchor points distributed approximately every 12 Kb along the BAC contigs (Supplementary Table 3).

### Assembly of a *de novo* physical map, tile path selection, and construction of homoeolog specific pseudomolecules

A *de novo* genome assembly was performed with the BAC fingerprint dataset using ultra-stringent assembly parameters (see Methods) to group nearly perfect overlapping BACs into contigs with no mismatch band overlaps to avoid cross assembly of homeologous sub genomes. The *de novo* TM-1 BAC physical map (herein referred to the Physical Mapping Initiative, PI) assembly consists of 92,391 validated BAC fingerprints, of which, 82,816 amassed 7,906 contigs and 9,575 singletons. The total estimated contig assembly length is 3,134 Mb. Contig sizes range from 45 Kb to 3.8 Mb, with a mean contig length of 396 Kb, and an N_50_ contig length of 308 Kb (Table 1). The *de novo* physical map was further integrated with the ~179,000 corresponding BES pairs and aligned to the high-quality *G. raimondii* (Gr) reference genome assembly^6^ for sub genome binning, and subsequent sub genome reassembly and pseudomolecule construction (See Methods). In the PI, the A sub genome of TM-1 assembled as 5,298 BAC contigs that collectively span 1.9 Gb; the contig sizes range from 46 Kb to 3.8 Mb with a mean length of 369 Kb and N_50_ of 287 Kb (Table 1). A minimal tile path was selected for the A sub genome that consists of a total of 12,389 clones. The D sub genome of TM-1 assembled as 1,998 BAC contigs that collectively spans approximately 1.1 Gb; the contig sizes range from 54 Kb to 4.3 Mb, with a mean length of 558 Kb and N_50_ of 419 Kb, and an MTP of 8,609 clones (Table 1). Manual review of the *G. hirsutum* (TM-1) sub genome physical map assemblies confirmed that the BACs are relatively evenly distributed within and between contigs, suggesting adequate genomic distribution and homeologous sub genome partitioning (Supplementary Figure 3). BAC contig pseudomolecules were prepared for each A and D sub genomes, which range in size from 48–96 Mb for PI A and 44–73 Mb for PI D. Average pseudomolecule lengths were ~77 Mb for PI A and ~59 Mb for PI D_,_ and resulting in PI A_,_ lengths that were ~30% larger than their homeologous counterpart in the PI D sub genome (Table 2 and Supplementary Table 4). When compared to the *G. raimondii* reference assembly (Gr), the PI A sub genome pseudomolecules were generally larger (~24%), while the PI D sub genome pseudomolecules were generally similar in size to the orthologous regions in the *G. raimondii* assembly^6^ (Supplementary Table 4). The final physical maps can be accessed at: (https://www.genome.clemson.edu/cgi-bin/cotton_gb/gbrowse/Gossypium_hirsutum/)

**Table 1:** *De novo* physical map assembly statistics.

	De novo	A-subgenome	D-subgenome
Total fingerprints (validated)	92,391	58,485	33,906
BACs in contigs	82,816	46,014	33,022
Average number BACS/contig	10	9	17
No. Contigs	7,906	5,298	1,998
No. Contigs (anchored)		2,370	1,000
No.Contigs (unplaced)		2,928	998
No. Singletons	9,575	12,471	884
Total Contig length (Mbp)	3.1	1.9	1.1
Longest contig (Mbp)	3.8	3.8	4.3
Shortest contig (Kb)	45	46	54
Mean contig length (Kb)	396	369	558
N50 contig (kb)	308	287	419
Minimal Tile Path (no. clones)		12,389	8,609

**Table 2:** *G. hirsutum* TM-1 PI pseudomolecule lengths.

*G. hirsutum* pseudomolecule	Length (bp)
PI A1	90,616,590
PI A2	60,403,230
PI A3	86,809,905
PI A4	77,945,040
PI A5	48,044,880
PI A6	85,263,480
PI A7	72,898,790
PI A8	96,529,545
PI A9	62,615,205
PI A10	81,184,050
PI A11	79,980,840
PI A12	76,012,335
PI A13	81,823,500
PI D1	73,209,284
PI D2	64,675,878
PI D3	46,884,792
PI D4	44,842,490
PI D5	71,793,353
PI D6	63,336,944
PI D7	53,430,680
PI D8	62,651,828
PI D9	44,173,004
PI D10	62,620,504
PI D11	63,570,550
PI D12	56,052,445
PI D13	60,522,067

### Alignment of BAC physical and BES linkage maps to *G. raimondii* and two *G. hirsutum* draft sequence assemblies

**Figure 1:**
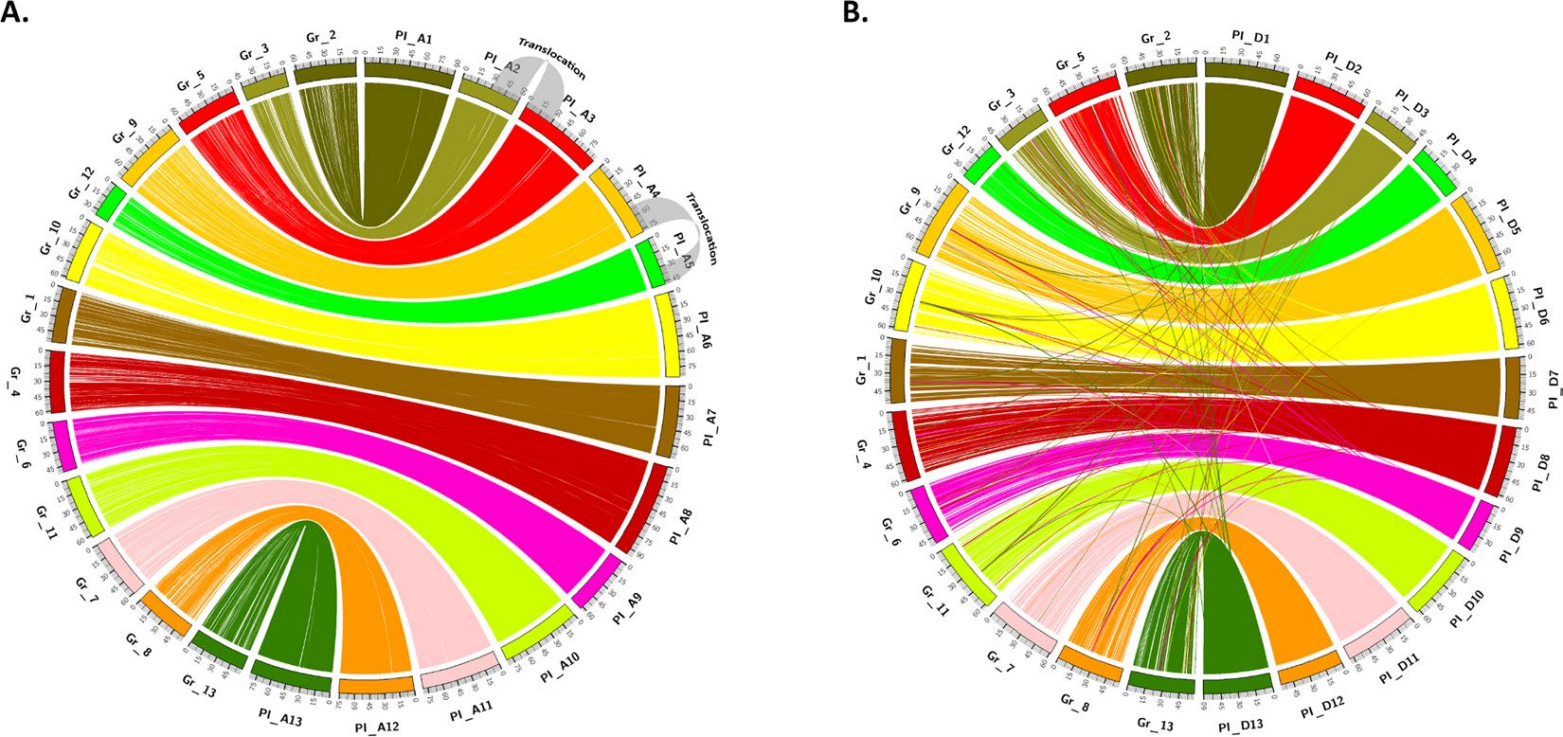
(**A**) Alignment of the *G. hirsutum* A-sub genome physical map pseudomolecules (PI) to the *Gossypium raimondii* JGI reference genome assembly (Gr). Two known translocations (A2/A3 and A4/A5) in the cotton tetraploid A-sub genome are highlighted by the outer grey links on the ideograms. (**B**) Alignment of the *G. hirsutum* D-sub genome physical map pseudomolecules (PI) to the *Gossypium raimondii* JGI reference genome assembly (Gr). Colored ribbons connect contiguous blocks of at least 6 BAC-end sequences with at least 95% sequence identity between the BAC-map contigs and the reference genome assembly.

**Figure 2:**
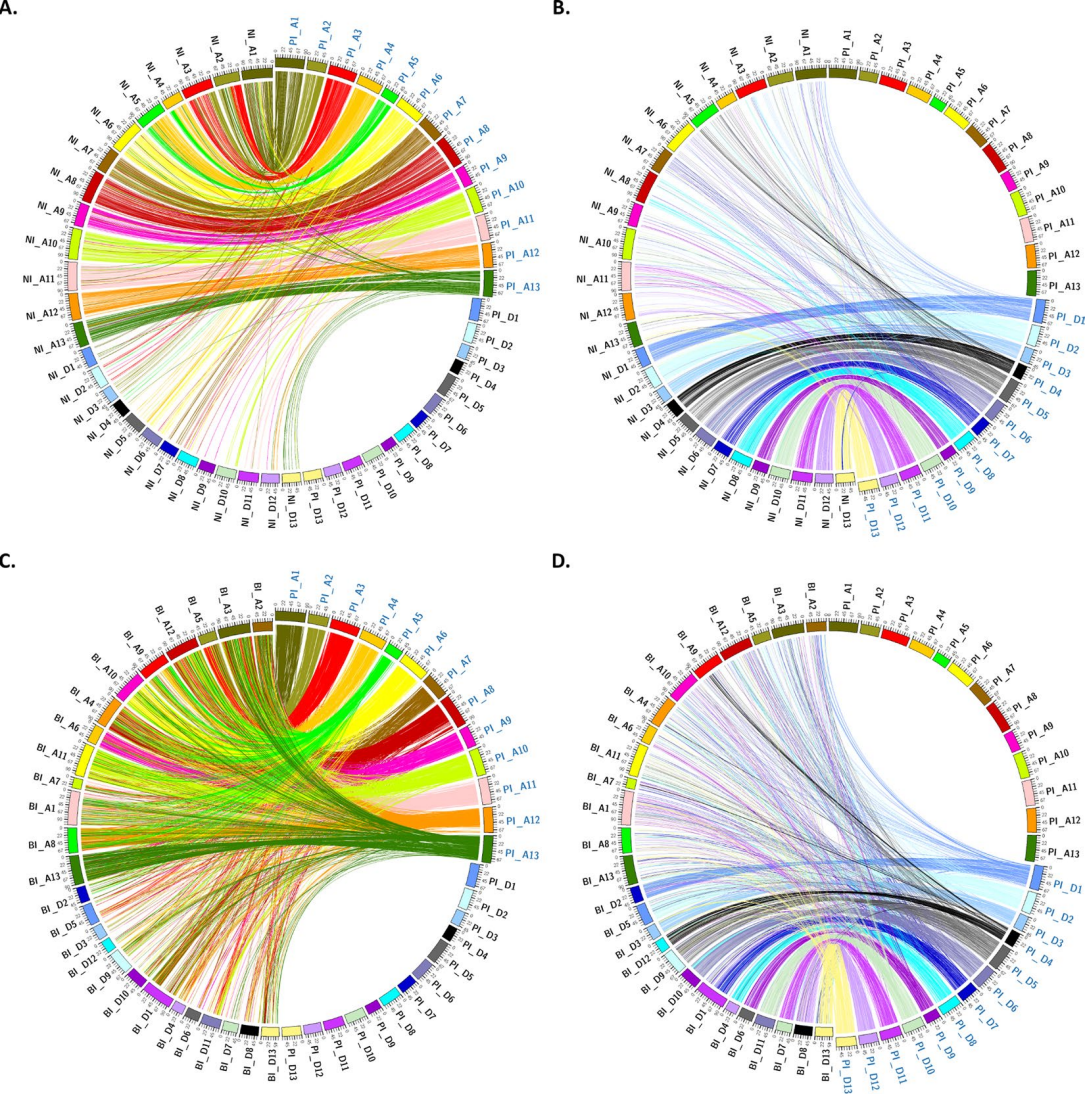
(**A**) Alignment of the *G. hirsutum* inbred TM-1 A-sub genome physical map pseudomolecules (PI) to the International Cotton Sequencing Consortium draft sequence (NI). (**B**) Alignment of the *G. hirsutum* D-sub genome PI pseudomolecules to the corresponding NI draft assemblies. (**C**) Alignment of the *G. hirsutum* A-sub genome physical map pseudomolecules (PI) to the Institute of Cotton Research (BI) draft sequence. (**D**) Alignment of the *G. hirsutum* D-sub genome physical map pseudomolecules (PI) to the corresponding BI draft sequences. Colored ribbons connect BAC physical map contigs to the respective reference genome assemblies with alignment criteria of 98% identity and a cluster of at least 6 contiguous BAC end sequences.

The *G. hirsutum* sub genome physical map pseudomolecules were aligned independently to the *G. raimondii* (Gr) reference genome assembly^6^ as a means to assess assembly completeness and colinearity. Using stringent alignment criteria and filters, we found that the tetraploid physical maps are largely colinear with the published diploid progenitor reference sequence (Fig. 1A and B, and Supplementary Figure 3). Similar to our findings of the overall pseudomolecule size comparisons (Supplementary Table 4), *G. hirsutum* TM-1 PI pseudomolecules are larger than the Gr scaffold assemblies with colinear links that anchor the tetraploid pseudomolecules from end-to-end for each of the pseudomolecules (Fig. 1A and B). The colored links also illustrate the density of BAC-end sequence clusters in each respective sub genome physical map, implying successful sub genome partitioning. The previously discovered genomic translocation events on chromosomes A 2/3 and A 4/5^34^ were also discovered, (Fig. 1A), in addition to large blocks of high sequence identity.  With strict alignment and filter criteria, the PI BAC map was aligned to the currently available *G. hirsutum* draft genome assemblies, the Novogene Bioinformatics Institute-International Cotton Sequencing Consortium (NI)^21^ and the Beijing Genomics Institute – Institute for Cotton Research (BI) assembly^20^, which revealed similarities and differences (A-D). By comparing alignments of the PI A-sub genome with the NI and BI A/D-sub genomes a, we found the most congruent alignments to the NI (Fig. 2A and B) draft assembly, with one-to-one matches aside from homeologous matches with the alternate subgenome (Fig. 2A, links to NI A01 - NI A13). Alignment of PI BAC assembly to the BI draft assembly resulted in a significantly fewer number of shared colinear alignments, in which a very large number of BES cluster matches (including blocks of six contiguous BAC-end sequences) aligned to many genomic segments distributed throughout both draft sub genomes, suggesting drastically different assembly builds (Fig. 2C and D). Alignment to NI assembly had the most congruent matches, but a significant number of gaps were observed (Fig. 2B), while alignment to the BI assembly had the most match elsewhere bins (Fig. 2C and D), suggesting possible incorrect sub genome placement either in the physical map and NI assemblies or the BI assembly. The physical map is publically available at: (https://www.genome.clemson.edu/cgi-bin/cotton_gb/gbrowse/Gossypium_hirsutum)

### Validation of physical map using BES-associated SNP linkage mapping and BAC-FISH

Using BES, a set of SNPs was identified^35^ and included as part of the CottonSNP63K Infinium array^36^. The homeologous chromosome pair A12/D12 (which will be used throughout the manuscript, but have also been referred to as Chromosomes 12 and 26) were selected for further investigation. A total of 310 BES-associated SNP markers were integrated in the published interspecific F_2_ map^36^ and their placement in the whole genome linkage map were compared to the placement of their corresponding BAC in the physical map. This analysis showed that the chromosome assignment in PI is largely correct with only a small number of markers being assigned to different homeologous pairs (10/310 = 3.2%), i.e., not A12 or D12. The majority of the BAC contigs (278/310 = 89.7%) could also be assigned to the predicted sub genome. To assess in more detail the chromosome specific quality of the physical map, the genotypes for 310 markers from the CottonSNP63K were combined with an additional 108 markers for chromosomes A12 and D12 to develop a higher density BES-SNP linkage map (see Methods). Chromosome A12 resulted in 100.78 cM in total length with 209 markers representing 135 unique recombination bins and D12 was 139.84 cM in total length with 199 markers representing 86 unique recombination bins (Supplementary Tables 5 and 6). High co-linearity was observed between these newly produced maps and the previously determined chromosome maps with all segregating markers off the array, with an R^2^ of 0.9962 and 0.9946 for the two chromosomes, respectively.

**Figure 3:**
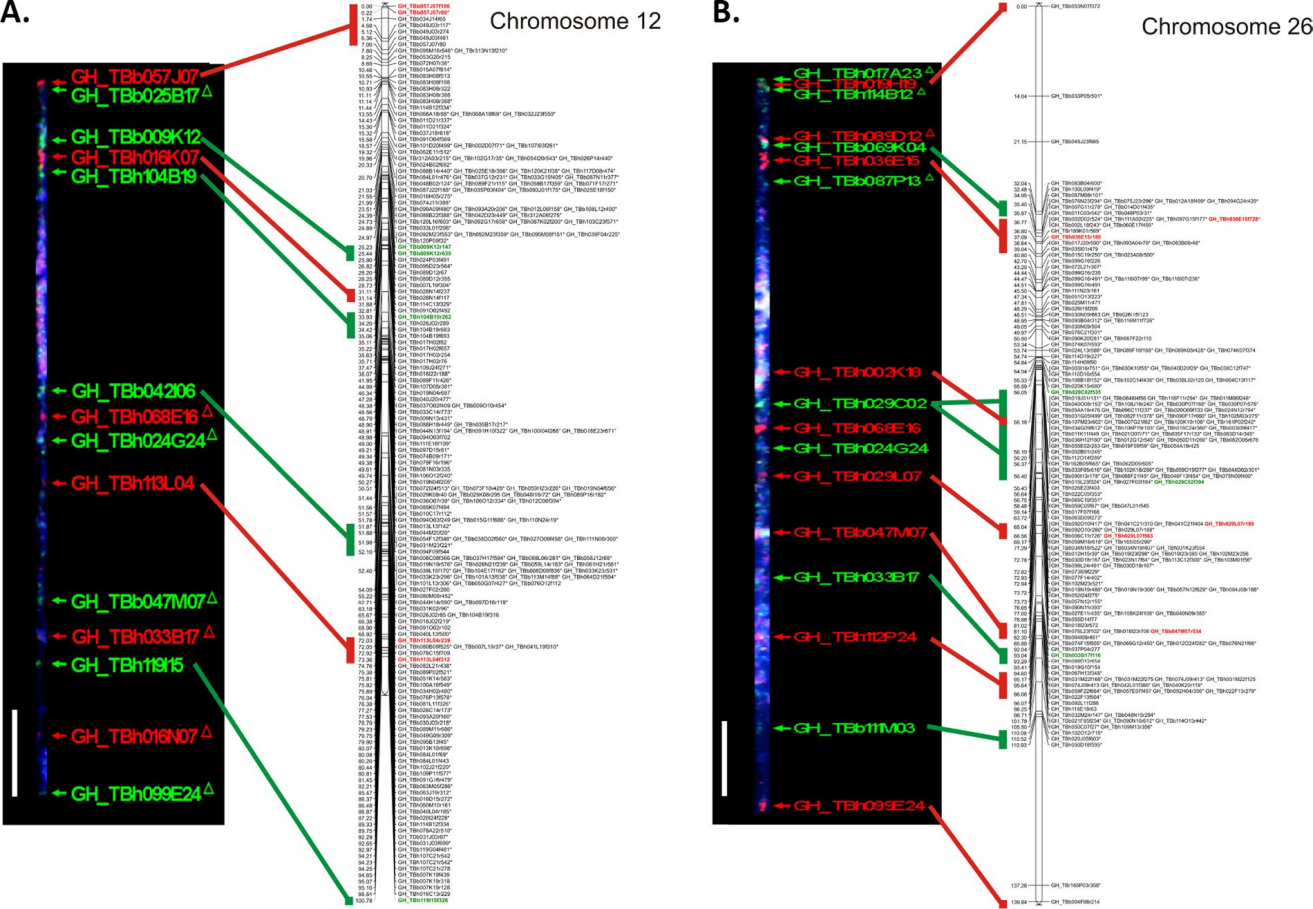
Relative positions of BACs from chromosomes 12 (A12) and 26 (D12) in cytological versus linkage maps. Cytological positions were determined by concomitant FISH of multiple BACs in chromosome-specific multi-BAC probe cocktails to spreads of meiotic pachytene bivalents from *Gossypium hirsutum* var. TM-1. Images of A12 and D12 bivalents are from two cells. White bars represents 10 µM. Linkage map positions for SNPs associated with end sequences of BACs in the respective BAC contigs are demarcated by vertical colored bars. (**A**) *G. hirsutum* bivalent and linkage group for chromosome A12. (**B**) *G. hirsutum* bivalent and linkage group for chromosome D12.

We selected 26 BACs from pseudomolecules of homeologous chromosomes A12 and D12 that were distributed throughout the physical map, and placed them on *G. hirsutum* pachytene bivalents using fluorescent *in situ* hybridization (FISH) (Fig. 3A and B). Twelve of the BACs mapped to the short arms (top of Fig. 3); while 14 BACs mapped to the long arms (bottom of Fig. 3A and B) of chromosomes A12 and D12. Several BACs hybridized to both homeologous chromosomes due to large amounts of shared sequence between homeologous chromosomes (Supplementary Table 7). Of 10 BACs that were associated specifically with 21 SNPs included in the linkage mapping, FISH enabled sub genome placement of 5 BACs according to differential signal intensity; and in every case the placement was corroborated by placement of the BES-associated SNPs in the linkage map. For the other 5 BACs, all were validated to be in the correct sub genome in the physical map using the BES-associated SNPs. A BAC-specific SNP was not identified for the remaining 16 BACs in the FISH map, but 14 of the 16 occur in BAC-contigs containing one or more mapped BES-associated SNPs. Within this group, 9 BACs had congruent placement information in the physical map, FISH and linkage mapping. Overall the comparative analysis shows high levels of agreement (near 100%) between BAC FISH, BES-SNP linkage mapping, and physical maps, where within the validated set here, the associated SNP linkage map placement and signal intensity placement with FISH agreed 100% of the time. Out of the 26 BACs that were included in the FISH map, only 2 were identified to be in different sub genomic position relative to the physical map.

### Sequencing of homeologous chromosome pair A12/D12 using large clone pools

Using the physical map, we tested MTP clone-based sequencing strategies of homeologous chromosome pairs A12 and D12. The large-pool sequencing approach included 463 overlapping MTP BACs from PI A12 (15 distinct clone pools) and 347 BACs from PI for D12 (12 distinct clone pools). In brief, ~30 overlapping tile path BACs per pool were prepared with a single index and sequenced using a standard Illumina shotgun sequencing approach (Table 3) (see Methods). The sequence assemblies of individual pools ranged from 2.7 to 4.4 Mb in length, with an average of 3.7 Mb, and the number of scaffolds ranged from 40 to 400 with an average of 251 (Supplementary Table 8a/8b). Total pseudomolecule sizes were 42 Mb and 38 Mb for chromosomes A12 and D12, respectively (Supplementary Table 9). Alignment of the A12/D12 pseudomolecules to the *G. raimondii* orthologous scaffold 08 revealed a significant amount of structural colinearity (Supplementary Figure 4A and C). Gene prediction of these two pseudomolecules identified 1,746 and 2,009 genes, respectively (Supplementary Tables 10,11). The A12 and D12 pseudomolecules each had a total of 3,112 and 3,711 annotated repeat sequences, respectively (Supplementary Table 14). LTR, *Gypsy*, and *Copia* repeats were the most abundant, with fewer distributions of DNA and other repeat types throughout the assemblies (Supplementary Figure 5, Supplementary Table 14). A12 and D12 psuedomolecule data is available at NCBI under the BioProject Accession PRJNA411996 under accessions CP023742, CP023745).

**Table 3:** Large and small clone pool sequencing of homeologous chromosome pairs A11/D11 and A12/D12.

Phys. Map Chromosome	No. MTP BACs	No. scaffolds	Estimated length (FPC)	Actual length (bp)	Gr. scaffold	Gr. length (Mbp)
PI A11	441	209	63,681,390	51,733,654	7	63.6
PI D11	407	116	63,641,115	42,228,869	7	63.6
PI A12	465	3244	76,012,335	40,542,802	8	57.1
PI D12	347	3223	56,052,360	36,445,133	8	57.1

### Sequencing of homeologous chromosome pairs A11 D11 using small clone pools

**Figure 4:**
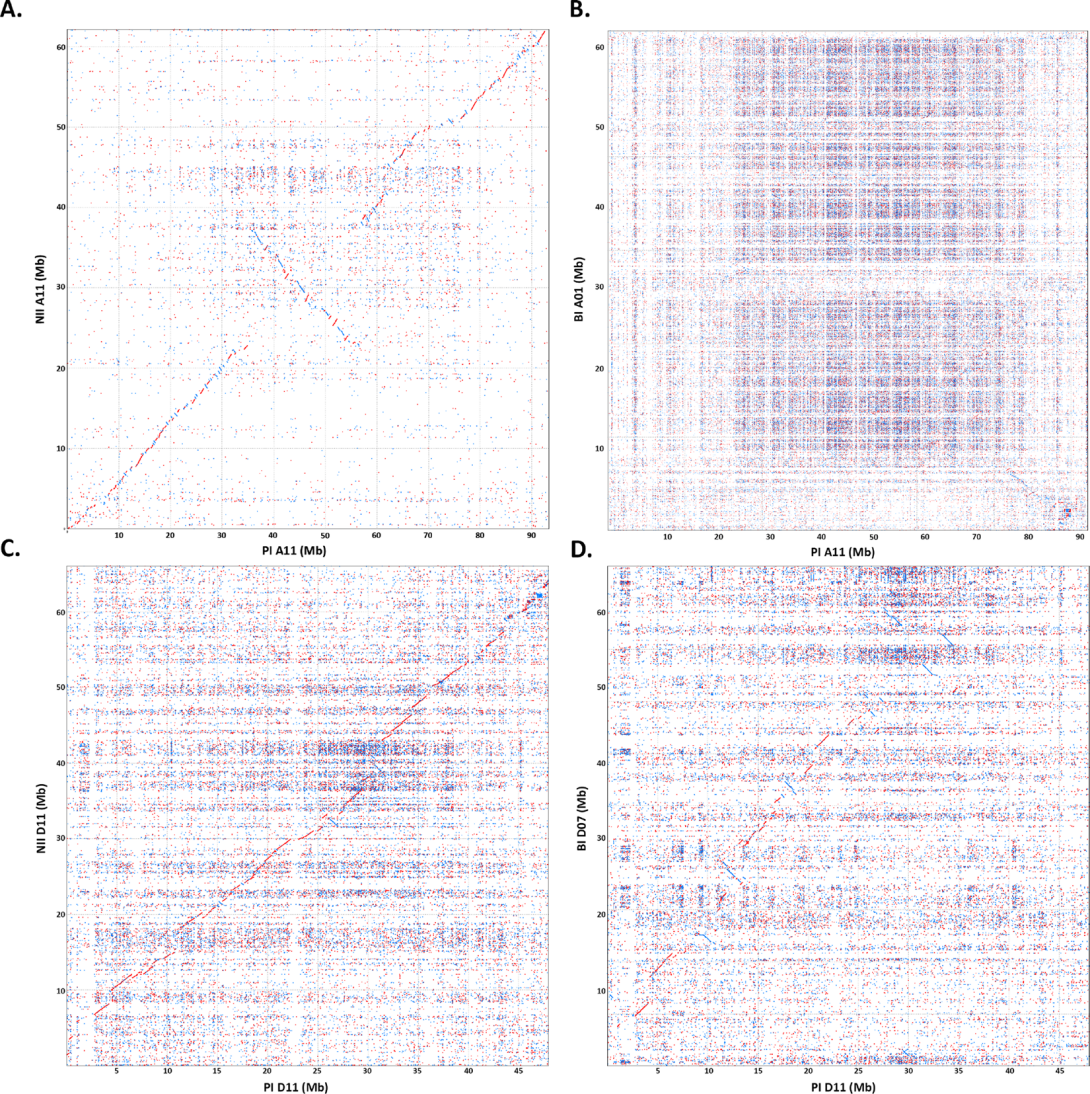
(**A**) Dot plot of the pilot BAC pseudochromosome (PI A11) as aligned to the corresponding NI A11 draft pseudomolecule. (**B**) Dot plot of the pilot BAC pseudochromosome (PI A11) as aligned to the corresponding BI A01 draft pseudomolecule. (**C**) Dot plot of the pilot BAC pseudochromosome (PI D11) as aligned to the corresponding NI D11 draft pseudomolecule. (**D**) Dot plot of the pilot BAC pseudochromosome (PI D11) as aligned to the corresponding BI D07 draft pseudomolecule.

The results from the large-clone pool chromosome assemblies generally agree well with the *G. raimondii* reference genome, however the presence of gaps and unincorporated scaffolds, suggest an improvement for pool design and size. In a complementary strategy, we assessed sequencing another pair of homeologous chromosomes A11/D11 (which will be used throughout the manuscript, but have also been referred to as Chromosomes 11 and 21) using a range of clones per index (1, 2, 4, 8, or 18 clones). This approach was used to sequence 441 MTP BACs for A11 and 407 MTP BACs for D11, respectively. Out of 848 clones, only 22 clones (2.6%) could not be assembled into a single consensus assembly, which could result from low sequencing coverage or clone dropout during DNA isolation, pooling, or library preparation. Consensus molecule sizes ranged from 28 Kb to 323 Kb, with an average BAC insert assembly size of 148 Kb, which is consistent with the BAC insert estimations. BAC scaffolds were aligned to the physical map and assembled as pseudomolecules with final scaffold counts of 209 and 116 for chromosomes A11 and D11, respectively. The final pseudomolecule lengths were ~51.7 Mb for A11 and ~42.2 Mb for D11, with BAC scaffold sizes ranging from 53 Kb to 1,099 Kb, and a mean scaffold size of 247 Kb for chromosome A11 (Supplementary Table 9). Pseudomolecule D11 scaffold sizes were slightly larger on average, and ranged from 106 Kb to 1,011 Kb, with a mean scaffold size of 364 Kb. Gene prediction identified a total of 2,246 and 2,394 gene sequences for these two chromosomes, respectively (Supplementary Tables 12 and 13). A search of the Gene Ontology (GO) terms for defense revealed 142 genes in chromosome A11 and 190 in chromosome D11 (Supplementary Tables 12 and 13), consistent with these two chromosomes known to harbor defense related genes^37^. Alignment of both pseudomolecules to Gr scaffold 07 revealed longer segments of contiguity, suggesting a more contiguous, complete build than using larger clone pools (Supplementary Figure 4). Moreover, predicted annotated repeat content was higher in A11 than A12; and also higher in D11 than D12 (Supplementary Figure 4; Supplementary Table 14), which is likely an artifact of the sequencing and assembly strategy. With the longer contiguity of the first-pass assemblies of PI A11/D11 homeologous pair (relative to PI A12/D12), these assemblies were aligned to corresponding pseudomolecules of NI (A11/D11) and BI (A01/D07) draft genome assemblies (Fig. 4A–D). The most congruent alignments were observed between the PI and NI D11 pseudomolecules (Fig. 4C). The PI and NI A11 alignments were also fairly colinear, but displayed a macro inversion and many small inverted segments (Fig. 4A) that resemble similar ordering. Alignments of the PI D11 pseudomolecule to the BI D07 pseudomolecule was ambiguous in many places, with non-contiguous contigs and short match segments (Fig. 4D). Interestingly, alignment of the PI A11 pseudomolecule to the BI assembly failed to produce any high-identity matches (Fig. 4B). A11 and D11 psuedomolecule data is available at (NCBI under the BioProject Accession PRJNA411996 under the accessions CP023743, CP023744).

### Identification of genes abounding public QTL intervals

Using the homeologous chromosome sequences, we searched for genes abounding public qualitative trait loci (QTLs) on chromosomes A11/D11 and A12/D1, respectively, using the public databases, such as (http://www.cottongen.org/; http://www.cottonmarker.org/; http://www.ncbi.nlm.nih.gov/). Using the homology-based annotation, several candidate genes were identified that may be involved in important traits such as nectariless, naked seed, male sterility, root knot nematode resistance, fusarium wilt resistance, bacterial blight resistance, and reniform nematode resistance from *G. aridum*, (Table 4). Trait intervals that align to chromosome A12 include *nectariless* (*ne1* – interval 15.4 Mb), *naked seed* (*N1* – interval 8.7 Mb), and male sterility (298Kb) (Table 4, Fig. 4). For example, trait intervals for *ne1* and *N1* harbored 594 and 437 genes, respectively. The male sterile QTL was much smaller and contained only 12 genes. Of these 12 genes, we identified a Cytochrome b5 reductase, which is essential for a functional male gametophyte in *Arabidopsis* ^38^, a phosphoglycerate mutase-like protein, which has been determined to be crucial to pollen formation in *Arabidopsis* ^39^, a tandem array of rapid alkalinization genes, which act as a negative regulator of pollen tube elongation during development^40^, a tandem array of 3 L-gulonoactone oxidase genes, a Heat shock 70 gene that has been shown to lead to thermosensitive gametophytic male sterility in *Arabidopsis* ^41^ (Supplementary Table 10). Another small QTL interval contains the bacterial blight resistance genes that maps to chromosome D12 (Table 4, Fig. 4). The interval size was ~1.0 Mb and harbored 48 candidate genes (Table 4, Fig. 4, and Supplementary Table 11). Interestingly, we identified a cluster of 7 mannitol dehydrogenase genes, 6 copies of serine/threonine-protein phosphatases, 3 copies of a cystolic serine/threonine-protein kinase, and several other plant signaling and defense related elements (Supplementary Table 11). Furthermore, a marker interval of ~9.4 Mb was identified that harbors 669 genes for the root knot nematode resistance QTL on chromosome A11 (Table 4, Fig. 4). Among these genes, there are 11 cytochrome p450 genes, multiple arrays of transcription factors (including 3 WRKY elements), three heat shock genes, five NAC domain containing proteins, nine pentatricopeptide repeat containing proteins, ten zinc finger protein genes, and many others related to defense and host immunity (Supplementary Table 12)^42,43^.

**Table 4:** QTL markers aligned to BAC-based assemblies of A12/D12 and A11/D11.

				PI A12				PI D12			
Trait	Mapping chr	Flanking Markers	Type	chr12 start	chr12 stop	Interval size	No. genes	chr26 start	chr26 stop	Interval size	No. genes
*Nectariless (NE1)*	A12	TMB2789	SSR	8,393,934	8,393,919	15,458,589	594	1,808,409	1,808,393	1,668,601	178
		BNL2894	SSR	23,852,387	23,852,523			139,792	139,776		
*Nectariless (NE2)*	D12	NAU2170	SSR	24,690,757	24,690,614	347,984	34	13,319,068	13,318,937	9,185,618	244
		NAU1231	SSR	25,038,865	25,038,741			22,504,660	22,504,686		
*Naked seed (N1)*	A12	NAU3862	SSR	1,075,530	1,075,495	8,781,226	437	23,479,480	23,479,499	10,419,078	302
		MUCS0303	SSR	9,856,984	9,856,756			13,060,174	13,060,402		
*Male sterile*	A12	NAU1278	SSR	18,319,569	18,319,490	298,370	12	25,986,076	25,986,060	2,039,828	133
		NAU2096	SSR	18,618,136	18,617,939			23,946,057	23,946,248		
*Male sterile*	D12	NAU3163	SSR	3,913,858	3,913,690	13,928,054	468	N/A	N/A	N/A	N/A
		DPL0838	SSR	N/A	N/A			1,068,678	1,068,662	2,754,066	261
		BNL1227	SSR	17,841,990	17,841,912			3,822,762	3,822,744		
				**PI A11**				**PI D11**			
**Trait**	**Mapping chr**	**Flanking Markers**	**Type**	**chr11 start**	**chr11 stop**	**Interval size**	**No. genes**	**chr21 start**	**chr21 stop**	**Interval size**	**No. genes**
*Triosephosphate isomerase (biotic and abiotic stress)*	A11	MUCS0399	SSR	43,018,392	43,018,546	607,856	11	24,650,439	24,650,455	1,088,232	21
		NAU3703	SSR	43,626,232	43,626,248			25,738,686	25,738,671		
*Root knot nematode resistance*	A11	CIR0316	SSR	768,585	768,601	9,430,332	669	26,500,308	26,500,290	7,320,774	155
		pGH648	SSR	10,198,935	10,198,917			33,821,100	33,821,082		
*Fusarium race*	A11	MUSB0827	SSR	26,879,919	26,879,751	23,161,628	335	7,511,327	7,511,497	8,701,630	528
		BNL3592	SSR	50,041,685	50,041,547			16,212,976	16,212,957		
*Bacterial blight resistance*	D11	par0535	SSR	2127872	2127739	25,098,129	1,107	23,511,066	23,511,310	1,486,163	48
		BNL2805	SSR	27225858	27226001			24,997,986	24,997,229		
*Reniform-ari*	D11	TMB1871	SSR	29,682,832	29,682,853	10,467,767	219	4,966,397	4,966,377	3,350,416	290
		STV0067	SSR	19,215,050	19,215,065			8,316,798	8,316,813		

## Discussion

All of the species in the Angiosperm lineage have undergone multiple rounds of genome duplication and repeated polyploidization events^44^. Segmenting a complex plant genome into manageable pieces through BAC cloning, and reconstructing these BACs into a chromosomal context by physical and genetic mapping for tile path sequencing, has set a high standard for quality and contiguity in genome sequencing projects. Moreover, whole-genome physical maps and BAC libraries are physical and lasting genomic resources that have critical value as tools, such as in the positional cloning of genes and associated regulatory sequences. Genome assemblies using whole-genome shotgun sequencing are being released at an unprecedented rate, but the quality and utility vary dramatically. Even with the aid of the second and third generation technologies (or a hybrid between the two), the results are typically similar, consisting of many short contigs and scaffolds comprised of errors and misassemblies.

The sub genomes of polyploid plants are generally large and contain an extensive repeat content, which can exponentially confound genome assembly algorithms resulting in non-contiguous and incorrect assemblies. This is particularly true in the case of the *Gossypium* genus. Except for the extant D-genome species, *G. raimondii* ^6^, draft *Gossypium* genome sequences are largely fragmented and comprised of small contigs and scaffolds, which is seen for the extant diploid A_2_ genome species^5^, for allotetraploid Sea Island cotton^19^, and for Upland cotton (var. TM1) by two groups^20,21^. Based on our physical mapping alignments, one TM-1 sequence^21^ aligns better than the other^20^, but neither is close to the reference-quality assembly. In this study, we developed a new-generation hierarchical BAC-based sequencing platform for Upland cotton. This is the first report of a whole-genome physical map of a polyploid species where BAC fingerprint contigs are successfully anchored to their respective sub genomes in a pseudomolecule context. This physical map of Upland cotton examines and reduces the complexity of the genome that can be integrated into a reference-grade sequencing strategy to maximize assembly accuracy and contiguity.

To coalesce conventional BAC physical mapping and hierarchal sequencing with multiplex sequencing technologies, we assessed differing clone pool sequencing strategies that include large clone pools composed of MTP BACs that are overlapping (~25–30 clones per pool) and smaller, non-overlapping BAC pools (1–8 clones per pool). Interestingly, we found that by sequencing contiguous sub-genomic segments of ~3-4 Mb in length, the resulting assemblies were fragmented and composed of many contigs and scaffolds. This was more pronounced in the A-sub genome chromosome A12, than the D-sub genome D12 large BAC pool assemblies, likely due to peri-centromeric repeat expansions that contribute to the larger A-sub genome size (~1.5X)^5^, and lack of divergence in the local repetitive content of our BAC assemblies. Sequencing of smaller clone pools yielded much more contiguous assemblies (longer contigs and scaffolds). The small BAC pool sequencing strategy has demonstrated a remarkable flexibility and scalability in the development of a hybrid method integrating new and traditional approach to a reference grade genome assembly.

These new TM-1 BAC resources developed here for the inbred variety Texas-Marker 1 (TM-1), contain the longest reported BAC inserts (150 Kb+) for a *Gossypium* species, compared with existing *Gossypium* BAC libraries such as Acala-type cultivar Maxxa with an average insert size of 137 Kb^45^, the restoring line 0-613-2 R with an average insert size of 130Kb^46^, a BiBAC library comprised of an average insert size of 135 Kb^47^, *G. tomentosum* with an average insert size of 122 Kb^48^, and *G. herbaceum* var. *africanum* with an average insert size of 115 Kb^49^. These new TM-1 BAC resources are the most representative of the Upland cotton genome because inserts were generated with two complimentary restriction enzymes and a random shear approach to minimize restriction bias and maximize coverage.

Validation of the physical maps through multiple methods including BAC-FISH and linkage mapping have shown that syntenic positioning of physical map contigs and largely sub genome placement of the physical map is correct. Although there are some instances in which contigs currently placed in the D-sub genome should be placed in the A-sub genome based on linkage map data and vice versa. This occurrence is not unexpected if there exist sizable homeologous segments that have high degrees of sequence identity, as might be caused by related descents as well as through homeologous sequence conversion via nonreciprocal exchange events^5,22^. The vast majority of the physical map and syntenic relationships hold up across multiple validation methods, and indicates the robustness of the approach. High-density SNP mapping using BAC-end associated SNPs^50^ offers a promising strategy through which to detect inconsistencies in the physical map and to easily reposition BACs under this framework method.

The estimated sub genome physical map pseudomolecule assembly lengths were generally larger in the allotetraploid when compared to the *G. raimondii* genome scaffolds^6^, which are likely because of BACs harboring repeat expansions in the allotetraploid, or because of overestimations of length as a result of the consensus band algorithm. Interestingly, alignment of the sub genome physical maps to the recently published allotetraploid draft genome assemblies Nanjing Initiative (NI)^21^ and the Beijing Genomics Institute assembly (BI)^20^ yielded similarities from a global perspective, with distinctions that separate the two at a finer scale. This may be due to the fact that the BES used in the Nanjing assembly were generated with the BAC library published here, and the connecting links are long and dense enough to facilitate accurate scaffolding. Furthermore, the number of unincorporated bins relative to the BI assembly could be a result of more repeats accurately resolved in the BI assembly, making placement via BES difficult. Most importantly, many gaps and non-colinear matches were detected in the comparison with both draft assemblies, suggesting a need for an interface that can be used to systematically improve the allotetraploid cotton genome to reference-grade quality.

Homeologous chromosome pairs A11/D11 and A12/D12 represent sets of chromosomes with traits of interest to breeders with the former having several genes associated with pathogen resistance and the latter associated with morphological traits e.g. nectariless, naked seed and male sterility^51^. Identification of the DNA sequence for these genes is highly desirable so that DNA markers with greater resolution can be developed for breeding purposes and screening the USDA-ARS cotton germplasm collection (~10,000 accessions) for new and valuable alleles. As a proof of concept, we used our approach to sequence a first pass of these chromosomes. Notably, we were able to link functional annotations of several candidate genes to traits where the flanking SSR boundaries were relatively small^52^. For example, several of the A12 candidate genes within the boundaries on chromosome A12 could be associated with a male sterility trait^38^, Moreover, an investigation of the genes underlying the bacterial blight QTL on chromosome 21 revealed a cluster of 7 mannitol dehydrogenase genes, which have been shown to have a direct role in plant defense against pathogens^53^. We also identified a serine/threonine-protein phosphatase gene, which functions in stress identification and signaling of defense mechanisms^54^, and a cystolic serine/threonine-protein kinase involved in defense signaling^55^. The availability of these sequences, and eventually a high quality reference genome of tetraploid cotton, will allow for finer genetic mapping through the discovery of targeted DNA markers and fine mapping with larger mapping populations. DNA markers tightly linked or at the gene of interest will greatly enhance marker assisted breeding in cotton, as will the identification of markers that are chromosome specific. The potential power of this resource was demonstrated recently when Feng *et al.* ^56^ used the *G. raimondii* sequence to identify SNPs closer to two male sterility genes on Chromosome A12. The new tetraploid genome resource could take us another step closer to identifying the gene(s) and identify markers for breeding and cotton improvement.

## Conclusions

The Upland cotton TM-1 BAC resources integrated with a new-generation sequencing strategy will provide a platform for producing reference-grade genome sequence for allotetraploid cotton and other polyploid crops. This approach affords the ability to selectively sequence difficult genomic regions and to finish the genome, in terms of completeness. The physical map has been validated using BAC FISH and linkage mapping with SNP markers developed from BES. Furthermore, the physical map has been used to evaluate quality of whole genome-shotgun assemblies from several recently published draft sequences of *Gossypium* diploid and tetraploid species, which has ensured the accuracy and contiguity of complex genome assemblies for Upland cotton.

## Methods

### BAC library construction

The *G. hirsutum* genetic standard line Texas Marker-1 (TM-1) seeds were obtained from co-author David M. Stelly (seeds can be requested by email: stelly@tamu.edu) and were propagated in greenhouse conditions for this study. Prior to tissue harvesting, the seedlings were dark-treated for 24 hours to reduce carbohydrate synthesis and photosynthetic byproducts. Approximately 100 grams of young, expanding leaf tissue was harvested, rinsed two times in ddH_2_0, blotted dry, and immediately flash frozen in liquid nitrogen. The restriction-derived BAC libraries were constructed by preparing intact nuclei according to previously published methods^57^ with the following modifications: addition of 1% (w/v) soluble PVP-40 (Sigma-Aldrich), 0.1% (w/v) L-ascorbic acid (Sigma-Aldrich), 0.13% (w/v) sodium diethyldithiocarbamate trihydrate (DIECA, Sigma-Aldrich), and 0.4% beta-mercaptoethanol to the nuclei isolation buffer (NIB) right before use. Post nuclei isolation and plug washing, the nuclei plugs were subject to pre-electrophoresis as a first step to remove small DNA (<80 Kb) and positively charged elements that may contribute as enzymatic or cloning inhibitors, following the methods of Osoegawa *et al*.^58^ with the following modifications: Plugs were run at 1 s:4 s switch time for 2.5 hours at 4 V/cm and soaked in 10 mM Tris-HCl overnight, changing the buffer at least 3 times. To prepare high molecular weight BAC inserts, the plugs were macerated and partially digested (separately) with, *Hin*dIII and *BstY*I using standard methods. BAC insert size selections, ligations, and transformations were carried out according to the methods of Lou and Wing^57^.

### High Information Content Fingerprinting and BAC-end sequencing

High Information Content Fingerprinting (HICF)^59^ was carried out on each BAC clone individually. BAC DNA was purified following standard alkaline lysis miniprep methods^60^. Purified BAC DNA was digested with *Ban*I, *Hin*dIII, *Nhe*I, *Xho*I, and *Pvu*II and labeled with the SNaP-shot labeling kit (Applied Biosystems) following the procedures of^61^. Prior to capillary electrophoresis, 9 ul of Hi-Di formamide and 0.05 ul of LIZ1200 were added to each BAC clone. BAC restriction profiles were resolved on an ABI3730xl (Applied Biosystems) with a 50 cm array and the raw data processed for sizing quality with the GeneMapper software package (Applied Biosystems) and converted to digital fingerprints with FPMiner (Bioinforsoft). Vector bands, clones with less than 20 or more than 200 bands were removed and the remaining data were uploaded to FPC v9.4^62^ for contig assembly. Using surplus DNA remaining from fingerprinting, BAC-end sequences for each clone were collected by dye-terminator sequencing of the clone ends with the universal priming sites (T7 and M13) that flank the multi-cloning site of the BAC vector and BigDye version 3.1 (Applied Biosystems). Dye terminator products were collected on short-run mode on an ABI3730xl and a 35 cm array.

### *De novo* physical map assembly, sub-genome assignment, and pseudomolecule assembly

An initial *de novo* assembly of all the *G. hirsutum* BAC fingerprints was performed with the FPC software v9.4^62^ at high stringency (Sulston cutoff of 1e^−80^) with a tolerance setting of 3 to minimize sub-genomic cross assembly. Questionable clones (Q-clones) were removed with the DQ’er using a setting of 10% and an iteration of contig end merges was performed with the Ends-To-Ends function with a Sulston cutoff of 1e^−75^ with contig merge requirements of at least 40 consensus band (CB) matches and overlap with at least 2 end clones. An iteration of Singles-To-Ends was performed to incorporate singletons to contigs at high stringency with a Sulston cutoff of 1e^−75^, and another round of DQ’ing was performed at 10%. The resulting *de novo* assembly was integrated with the corresponding BAC-end sequences to include positional information and aligned to the *Gossypium raimondii* reference assembly^6^ with megablast^63^ with an expectation value of 1e^−100^ and the –F F parameter set. BAC contigs were assigned as “D” sub genome when at least 4 BAC-end sequences aligned with ≥95% identity and relative colinearity with the *G. raimondii* reference assembly was conserved. The remaining contigs were binned as the “A” sub genome. In each case (A/D), the sub-genome assigned fingerprints were reassembled independently at a Sulston cutoff of 1e^−75^ with subsequent DQ’ing at 10% with every iteration of Ends-to-Ends and Singles-to-Ends joining until a final Sulston cutoff of 1e^−50^ was reached. The final A and D sub genome physical maps were then realigned to the *G. raimondii* reference assembly^6^ using megablast^63^ with an expectation value of 1e^−100^ and the –F parameter set. Contigs were ordered and oriented according to the best match to the *G. raimondii* reference assembly and a pseudo-framework file was created according to clone name and reference genomic coordinates as the physical location. Contigs were assigned and ordered as chromosomes using the Ctg - > Chr function in FPC. Contigs were placed into pseudomolecules using an iterative approach by end joining using an e-value of 1e^−20^ if contigs overlapped slightly and were adjacent in placement to the D5 assembly. A minimal tile path (MTP) was selected for each A/D physical map where MTP criteria for clone overlap falls between 15 and 35 Kb and priority is given to the longest clones.

### Physical map validation – Linkage mapping and BAC-FISH

Chromosomes A12 and D12 were selected for additional validation. BAC sequences along the contigs for each chromosome were prepared, labeled, mixed in a cocktail and then the cocktail was hybridized to pachytene bivalents using florescent *in situ* hybridization technique. Intensity and position of signals were analyzed to determine sub genome placement along chromosomes. Interspecific SNPs developed using BAC-end sequences (Hulse-Kemp *et al*. 2015) were selected representing BACs across the contigs for A12 and D12 including BACs that were selected for BAC-FISH. Primers for KASP assays (LGC Genomics) were designed using BatchPrimer3 and diluted according to manufacturer instructions. Primer sets were screened on a panel containing 12 samples including TM-1 (Stelly Lab), TM-1 (USDA), *Gossypium barbadense* line 3-79 (x2), F_1_–3-79xTM-1 (x2), RIL01-04 (3-79xTM-1) and water non-template control (x2) according to manufacturer’s suggested PCR conditions. Plates were read using the Pherastar at 38, 44 and 50 cycles, then analyzed using the KlusterCaller program. SNP assays that produced three expected clusters in the case of a co-dominant marker or two clusters in the case of a dominant marker, were used to genotype a population of 118 F_2_ (*G. barbadense* 3-79 x *G. hirsutum* TM-1) individuals on the Fluidigm BioMark HD. Genotypes were called using Fluidigm SNP Genotyping Analysis software and converted to ABH format. Genotype data for the same 118 F_2_ individuals were obtained for BAC-associated SNP markers included on the CottonSNP63K array (Hulse-Kemp et. al. 2015). ABH data files from both genotyping technologies were then then linkage mapped using JoinMap 4.1^64^ using grouping LOD of 5.0, removal of identical markers from groups with 100% identity, and ordering with regression and Kosambi’s mapping function. Identical markers were reincorporated to generate the final linkage groups. The resulting linkage groups were compared with the BAC-FISH results. Linkage maps were oriented similarly to Hulse-Kemp *et al*.^36^.

### Large clone pool sequencing of A and D homeologous chromosome pairs A12/D12

A total of 400 and 341 BACs for homeologous A and D chromosome pairs A12 and D12 were manually arrayed into 96-well format and grown individually for 18 hours in standard Laurel Broth liquid media (ThermoFisher). Cultures of overlapping BACs (~30 BACs per set) as outlined in the results were manually combined and plasmids were isolated using an in-house midi-prep procedure^65^ designed to minimize *E. coli* host contaminating DNA. DNAseq libraries were prepared for each BAC pool using the NexteraXT library kit (Illumina) and sequenced on an Illumina MiSeq using a 2 × 250 PE read type to at least ~100X per BAC. Raw trace data was preprocessed with the Trimmomatic software^66^ to remove adapter sequences and low quality bases. Preprocessed pools were assembled independently with the Celera WGS  assembler v8.1 Release (http://wgs-assembler.sourceforge.net/wiki/index.php?title = Main_Page). Contigs were aligned to the corresponding A and D sub genome physical maps via the integrated BES and arranged as pseudomolecules with 50k N’s added between pools.

### Small clone pool sequencing of A and D homeologous chromosome pairs A11/D11

A total of 441 and 407 BACs for homeologous A and D chromosome pair A11 and D11 were manually arrayed into 96-well format and grown individually for 18 hours in standard Laurel Broth liquid media (ThermoFisher) supplemented with 7% glycerol, and stored at −80 °C. 96-well deep well blocks containing 1.2 mL of TB/CHL media were inoculated with 2 ul of each BAC clone glycerol stock. The plates were sealed with airpore tape, incubated, and shaken at 37 **°**C for 18 hours. BAC DNA was purified using the Beckman Coulter Genomics CosMcPrep purification kit on a Biomek FX robot (Beckman Coulter) followed by fragmentation using a 96-well Covaris AFA instrument. Each clone was fragmented to an average size of 500 base pairs. Barcoded Illumina libraries were constructed using Ovation rapid DR Multiplex system in 96-well format (NuGEN Technologies Inc.). All cleanups were performed using RNAcleanXP beads (Beckman Coulter). After adaptor ligation, all libraries were pooled and size selected on a 1.5% agarose gel, 400–1500 basepairs (SAGE Science). qPCR was carried out on the final library to determine cluster density prior to running on an Illumina HiSeq instrument (2 × 250 bp) in Rapid mode (768 clones per channel). To assemble the individual barcoded clones, 35,000 pairs of sequence reads were extracted and then filtered for *E.coli* and chimeras using a custom kmer-based chimera identification algorithm. The remaining reads were then screened for additional vector or contaminant. The resulting reads were assembled using phrap on large memory linux cluster nodes with modified parameters “-vector_bound 10 -new_ace -minmatch 20 -maxmatch 40 -minscore 40 -trim_qual 20”. After assembly, reads from single read regions and identified chimeras from phrap were removed. Projects are then reassembled, followed by a repeat of the read removal step and then reassembly. Cotton whole genome shotgun reads were then identified that localized to the clone assembly by blat. These reads were filtered for local repeats and then reassembled into the clone assemblies. Single reads that caused breaks in existing clone contigs were then removed and the project reassembled multiple times.

Each clone was then manually curated by an experienced finisher using the Hudson Alpha consed pipeline^67^ and clone viewing software (unpublished). For each clone, BAC ends were identified and tagged followed by marking of high quality discrepancies and reassembly. Local repeats were resolved by manual manipulation of reads and tearing of sequence contigs. High confidence joins were made across simple repeats and in repeat regions supported by read pair data. Before completion, final assemblies were reviewed by a second experienced finisher.

## Electronic supplementary material

Supplementary Dataset 1

Supplementary Dataset 2

Supplementary Dataset 3

Supplementary Dataset 4

Supplementary Dataset 5

Supplementary Dataset 6

Supplementary Dataset 7

Supplementary Dataset 8

Supplementary Dataset 9

Supplementary Dataset 10

Supplementary Dataset 11

Supplementary Dataset 12

Supplementary Dataset 13

Supplementary Dataset 14

Supplementary Figures
