# Supplementary material for: Sub genome anchored physical frameworks of the allotetraploid Upland cotton (Gossypium hirsutum L.) genome, and an approach toward reference-grade assemblies of polyploids
Source: Sci Rep. 2017 Nov 10;7:15274. doi: 10.1038/s41598-017-14885-w (PMC5681701; doi:10.1038/s41598-017-14885-w)
Supplement: Supplementary file 15 — Supplementary Figures [file 41598_2017_14885_MOESM15_ESM.pdf]

Supplementary Figures

Title: Sub genome anchored physical frameworks of the allotetraploid Upland cotton (*Gossypium hirsutum* L.) genome, and an approach toward reference-grade assemblies of polyploids

Authors:

Christopher A. Saski[1,a*], Brian E. Scheffler[2,a], Amanda M. Hulse-Kemp[3,a], Bo Liu[3], Qingxin Song[4], Atsumi Ando[4], David M. Stelly[3], Jodi A. Scheffler[5], Jane Grimwood[8], Don C. Jones[6], Daniel G. Peterson[7], Jeremy Schmutz[8*], and Z. Jeffery Chen[4,*]

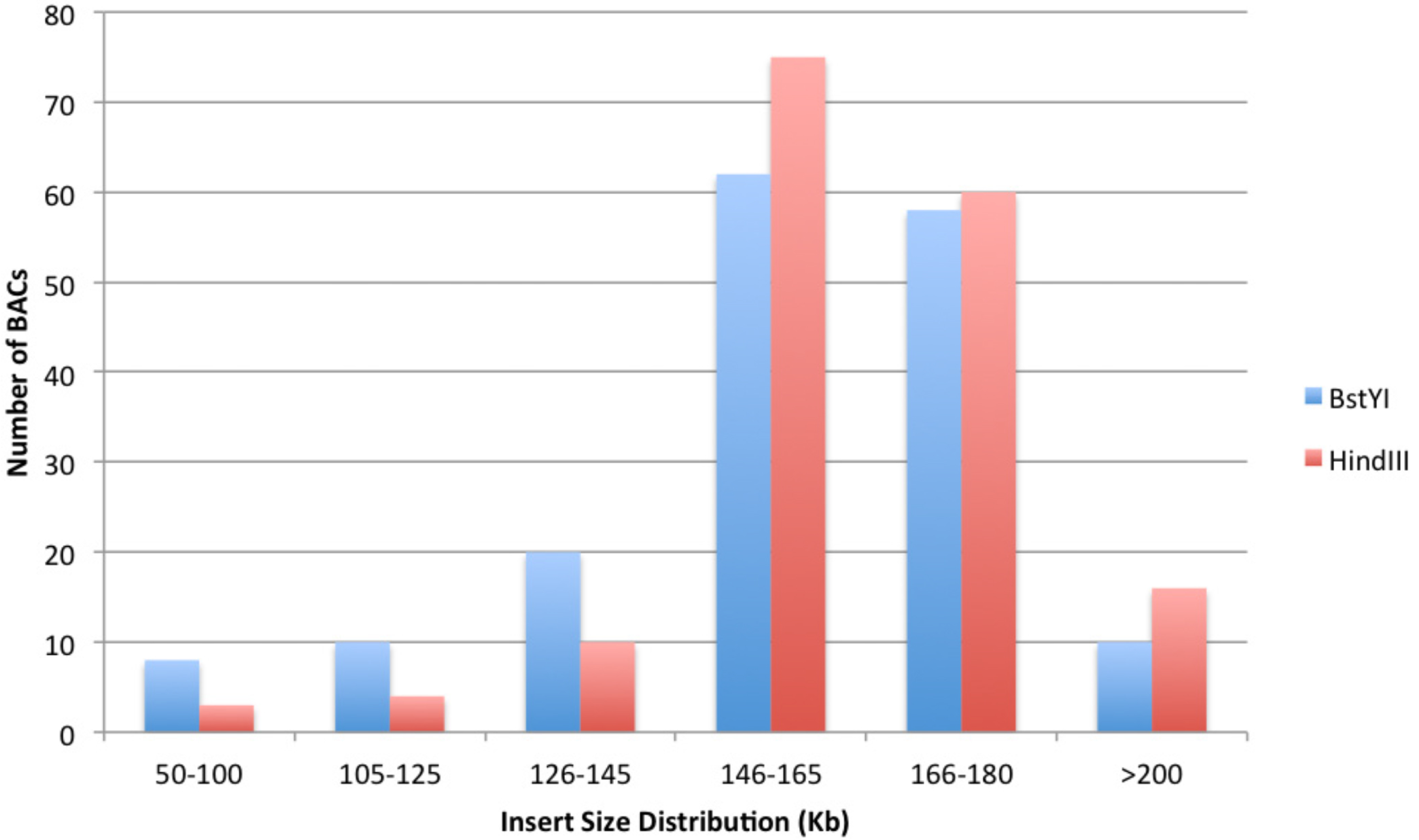

Figure S1. BAC insert size distribution of the restriction-derived TM-1 BAC libraries, GH_TBb and GH_TBh.

GH_TBb

GH_TBh

GH_TBr

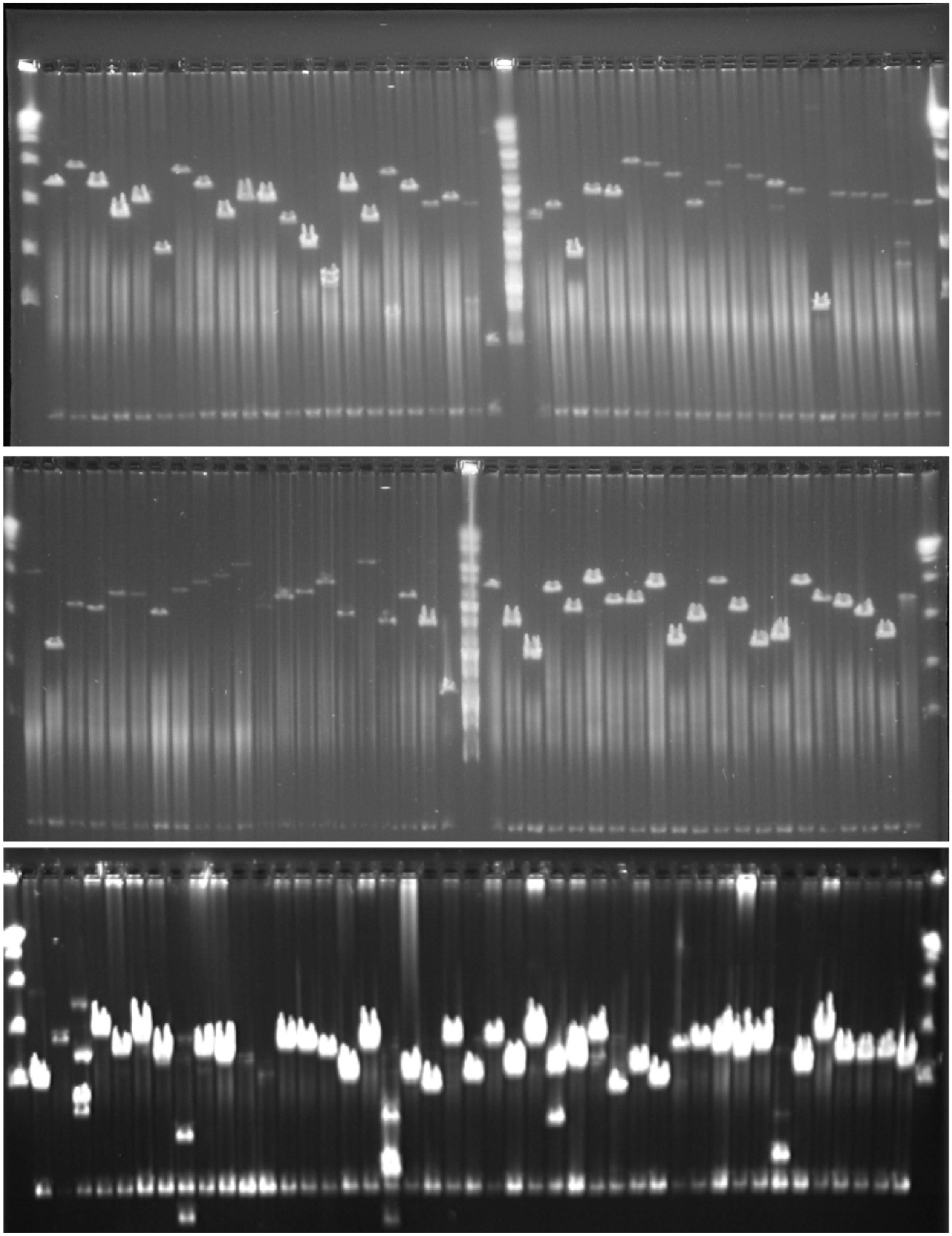

Figure S2. Figure S2 A-C. Pulsed-field gel electrophoresis of random *Not*I digested TM-1 BACs from GH_TBb, GH_TBh, and GH_TBr. The 2 outermost lanes contain Lambda ladder PFG marker (NEB) and the middle lane contains Lamba midrange PFG marker (NEB).

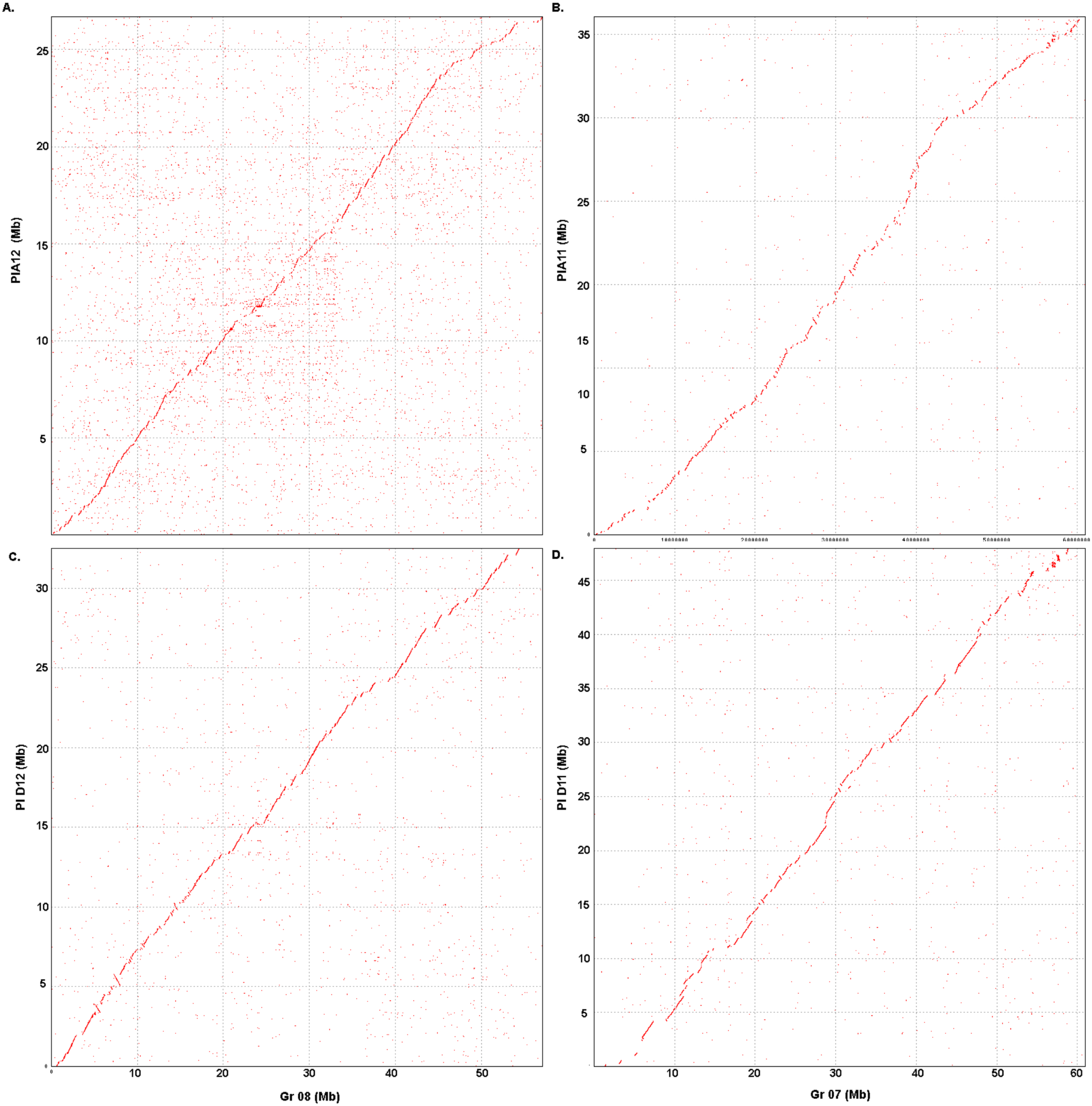

Figure S4. A. Dot plot alignment between *Gossypium hirsutum* chromosome A12 and *Gossypium raimondii* Chromosome 08. B. Dot plot alignment between *Gossypium hirsutum* D12 and *Gossypium raimondii* Chromosome 08.

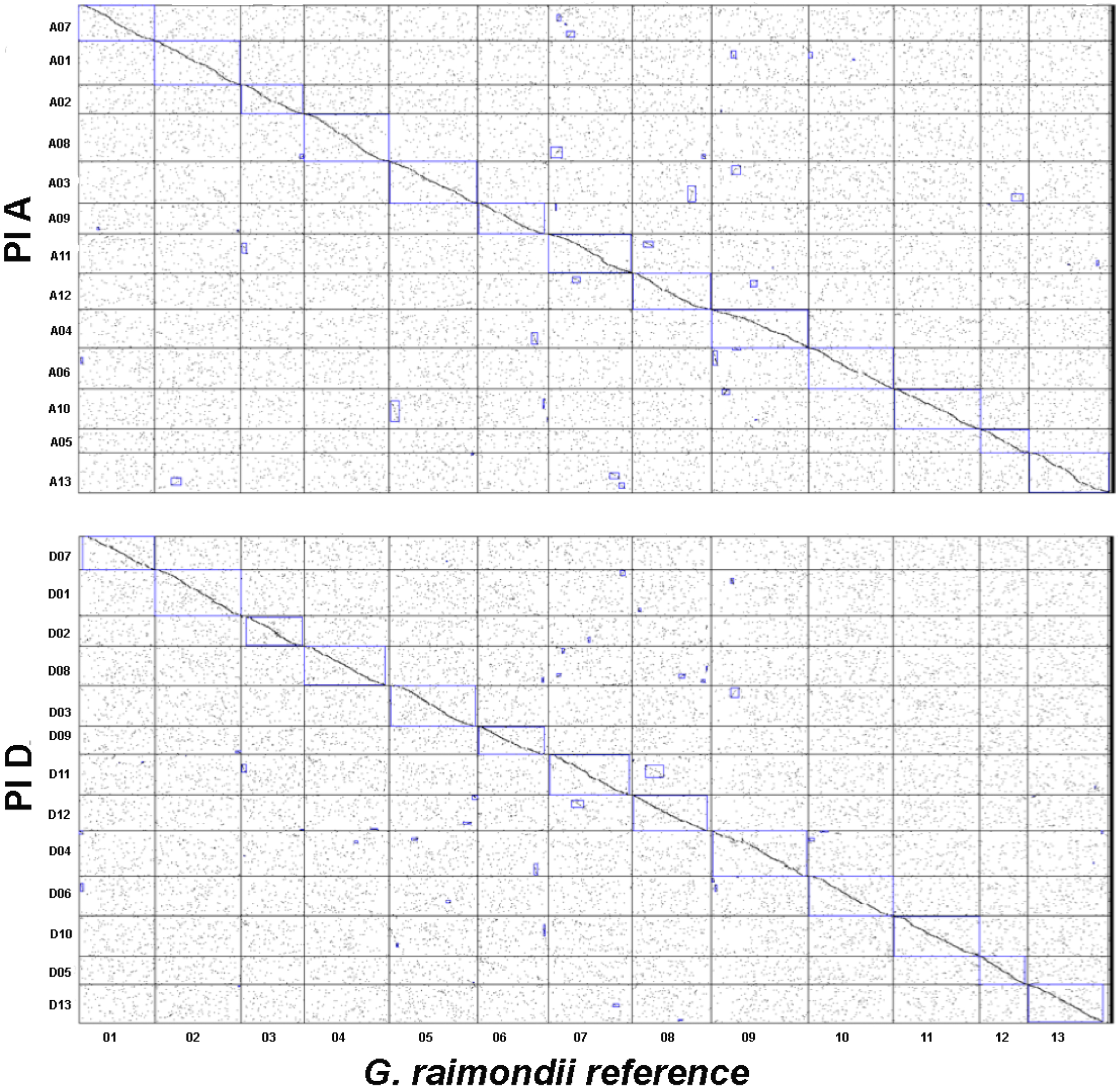

Figure S3. A dot plot alignment of the *G. hirsutum* TM-1 subgenome specific BES aligned to the *G. raimondii* extant diploid progenitor reference assembly. The BES alignments are relatively colinear and evenly distributed.

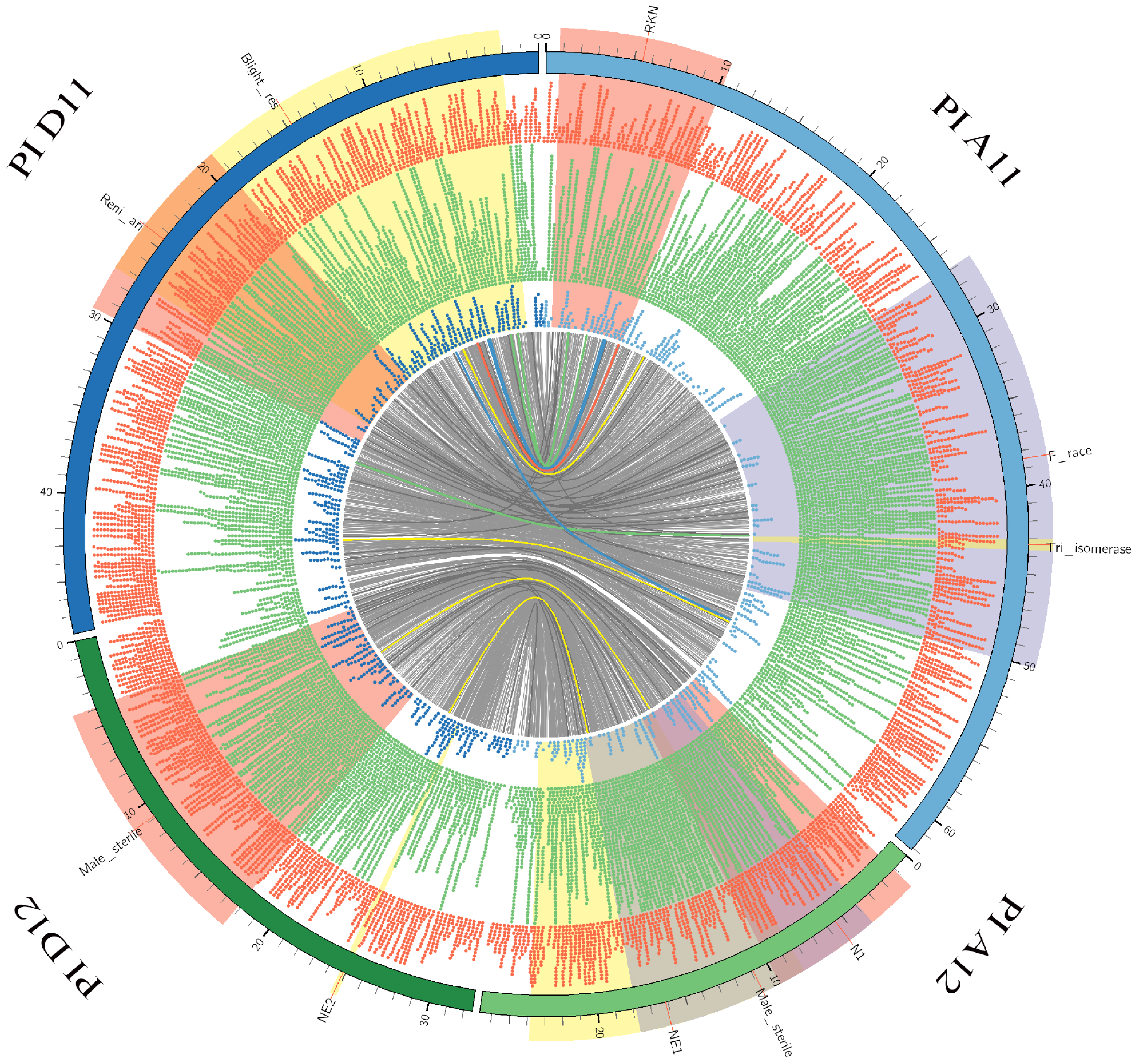

Figure S5. Pseudomolecule alignments of pilot chromosomes A11/D11 and A12/D12 as aligned as homeologous pairs by the inner links. Blue glyphs (internal) are retroelements, green glyphs are *Gypsy* elements, and red glyphs are *Copia* elements. Highlights denote SSR qtl boundaries of qualitative traits.
